# Couple experiences of provider-initiated couple HIV testing in an antenatal clinic in Lusaka, Zambia: lessons for policy and practice

**DOI:** 10.1186/1472-6963-13-97

**Published:** 2013-03-14

**Authors:** Maurice Musheke, Virginia Bond, Sonja Merten

**Affiliations:** 1 Zambia AIDS-related TB Research Project, University of Zambia, P.O Box 50697, Lusaka, Zambia; 2 Swiss Tropical and Public Health Institute, Socinstrasse 57, Basel CH-4002, Switzerland; 3 University of Basel, Petersplatz 1, Basel CH-4003, Switzerland; 4 Department of Global Health and Development, Faculty of Public Health and Policy, London School of Hygiene and Tropical Medicine, Keppel Street, London, WC1E 7HT, United Kingdom

**Keywords:** Antenatal clinic, Antiretroviral treatment, Couple HIV testing, Zambia

## Abstract

**Background:**

Couple HIV testing has been recognized as critical to increase uptake of HIV testing, facilitate disclosure of HIV status to marital partner, improve access to treatment, care and support, and promote safe sex. The Zambia national protocol on integrated prevention of mother-to-child transmission of HIV (PMTCT) allows for the provision of couple testing in antenatal clinics. This paper examines couple experiences of provider-initiated couple HIV testing at a public antenatal clinic and discusses policy and practical lessons.

**Methods:**

Using a narrative approach, open-ended in-depth interviews were held with couples (n = 10) who underwent couple HIV testing; women (n = 5) and men (n = 2) who had undergone couple HIV testing but were later abandoned by their spouses; and key informant interviews with lay counsellors (n = 5) and nurses (n = 2). On-site observations were also conducted at the antenatal clinic and HIV support group meetings. Data collection was conducted between March 2010 and September 2011. Data was organised and managed using Atlas ti, and analysed and interpreted thematically using content analysis approach.

**Results:**

Health workers sometimes used coercive and subtle strategies to enlist women’s spouses for couple HIV testing resulting in some men feeling ‘trapped’ or ‘forced’ to test as part of their paternal responsibility. Couple testing had some positive outcomes, notably disclosure of HIV status to marital partner, renewed commitment to marital relationship, uptake of and adherence to treatment and formation of new social networks. However, there were also negative repercussions including abandonment, verbal abuse and cessation of sexual relations. Its promotion also did not always lead to safe sex as this was undermined by gendered power relationships and the desires for procreation and sexual intimacy.

**Conclusions:**

Couple HIV testing provides enormous bio-medical and social benefits and should be encouraged. However, testing strategies need to be non-coercive. Providers of couple HIV testing also need to be mindful of the intimate context of partner relationships including couples’ childbearing aspirations and lived experiences. There is also need to make antenatal clinics more male-friendly and responsive to men’s health needs, as well as being attentive and responsive to gender inequality during couselling sessions.

## Background

A growing body of evidence shows that a large proportion of HIV infection occurs in marital or cohabiting relationships [1-4], for instance, 50-65% in Swaziland; 35-62% in Lesotho; and 44% in Kenya [4] and a high prevalence of discordant couples has been reported in some 12 sites of Eastern and Southern Africa [4]. Therefore, couple HIV testing has long been touted as essential for facilitating disclosure of HIV status in marital relationships [5,6]; adoption of risk reduction sexual behaviour [7-12]; uptake of treatment for prevention of mother-to-child transmission of HIV (PMTCT) [13,14]; and reduction in loss-to-follow up of women on treatment [14].

With a population of slightly over 13 million people, an estimated 14.3% of Zambians (aged 15–49 years) are living with HIV [15]; 69% of HIV-positive men and 49% of HIV-positive women may not be aware that they are infected [15]. Urban data on Zambia suggests that more than 60% of new infections occur within marriage or cohabiting relationships [4,16] and discordance rate is estimated at 11% [15,17]. Whilst more than 90% of women attending antenatal care services are tested for HIV - under an ‘opt-out’ strategy - only 10% of couples in Zambia have tested together for HIV [18].

The Zambian PMTCT protocol recommends the provision of couple HIV testing in antenatal clinics as part of HIV prevention, treatment and care [18]. However, there is little emprical evidence about how this is actually achieved and couples’ experiences of this testing strategy. As the World Health Organisation (WHO) has recently noted about couple HIV testing, “there are very few data… on adverse social and psychological outcomes such as those affecting quality of life, marital relationships or the risk of violence, including emotional abuse and gender-based violence [19]. Additionally, most studies on couple HIV testing in sub-Saharan Africa (SSA) have largely consisted of quantitative studies [12,14,20-25] and there have been calls for more studies to better understand issues related to its provision [5,19,23].

In view of this gap, this study explored couple experiences of couple HIV testing with a specific focus on the impact on treatment uptake, social support and adoption of risk-reduction sexual behaviour. The paper begins by giving an overview of couple HIV testing pathway in an antenatal clinic followed by an encapsulation of the strategies used by health care providers to promote it. Drawing on couple and provider experiences, we then present the effects of couple HIV testing on marital relationships and discuss policy and practical lessons.

## Methods

### Research location and context

The study was conducted in a high-density urban residential area of Lusaka, the capital city of Zambia. As a result of rural–urban migration that Zambia has witnessed particularly in the last three decades as people moved to the city in search of economic opportunities, the study setting comprises people from different ethnic groupings although two vernacular languages (namely *Bemba* and *Nyanja)* are the most widely spoken. While living conditions of the local people are mixed, the majority of residents are poor, mostly making a living within the informal sector of the economy.

Despite the diversity of ethnic groups, there are strong similarities in Zambian marriage practices [26] and inter-ethnic marriages are common. Payment of bride-price by the man’s family, locally called *lobola,* is common. There is a pattern of marriage taking place when a woman falls pregnant in casual relationships, partly to avoid a financial penalty charged by the woman’s family (locally called ‘damage’). This pattern of early pregnancies and subsequent marriages is reflected in the national fertility trend - 61% of women become mothers by the time they are 20 years old [15]. Since some of these marriages are often a ‘forced’ option and couples (particularly women) are young, in these unions, conflict, lack of intimacy and lack of social support are more evident. Although the majority of the marital relationships are supposedly monogamous, extra marital relationships and multiple concurrent non-spousal sexual partnerships are reported, often linked with hanging out in beer drinking places.

Health services in the area are mainly provided by a public sector clinic. The clinic has an estimated catchment population of over 150,000 and provides out-patient and in-patient health services. The clinic also provides HIV testing, PMTCT, family planning and reproductive health services; houses a couple HIV testing research project and a non-governmental organization that provides sexual education, nutritional counselling and psychosocial support to women and new mothers living with HIV.

### HIV testing pathway in the antenatal clinic

**Figure 1:**
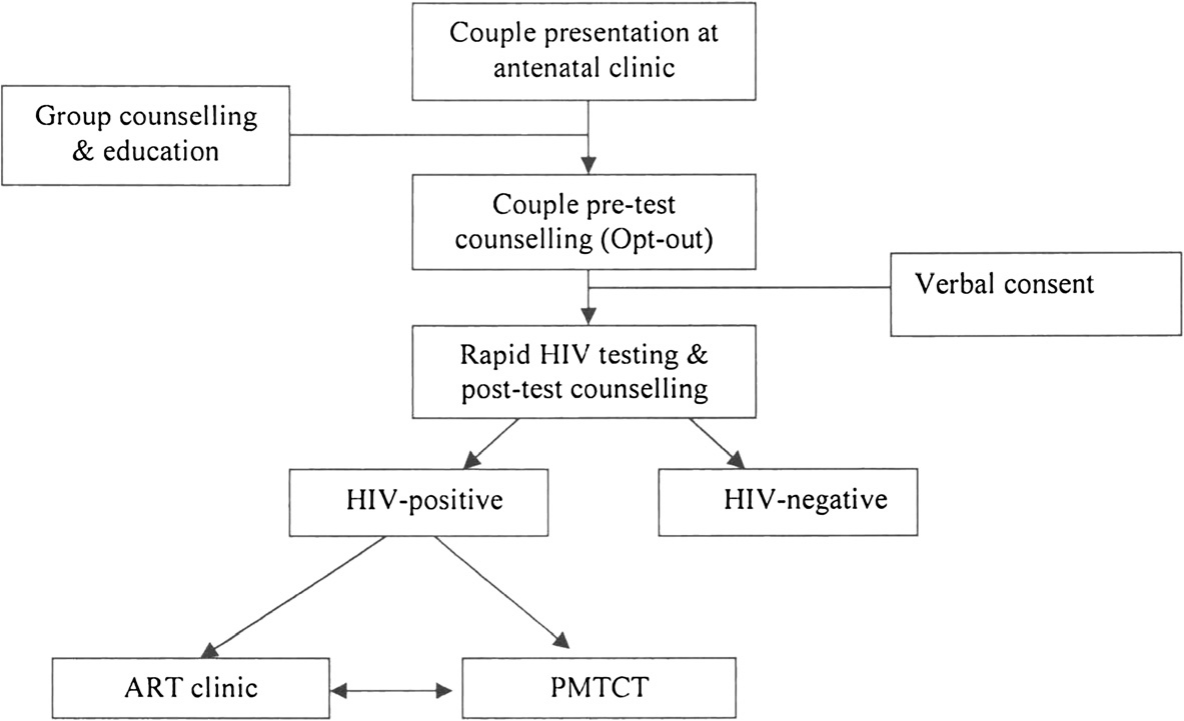
Couple HIV testing and treatment pathway at antenatal clinic.

When women present pregnant, they are booked for antenatal care. At the booking stage, married women are sometimes asked to go back home to bring their spouses for couple HIV counselling and testing. Group counselling and education is then provided focusing on inter alia, HIV transmission and prevention measures; the benefits of HIV testing including PMTCT; and implications of a positive HIV test result including the availability and provision of antiretroviral therapy (ART). To ensure that men - most of who are breadwinners for their families - do not stay long at the often congested antenatal clinic, couples are given preferential treatment. Group counselling and education is followed by individual and couple pre-test and post-test counselling. The men found HIV-positive are referred to the ART clinic (not based at the antenatal clinic) for further clinical assessments and enrolment into ART care while HIV-positive women continue to receive, inter alia, PMTCT care within the antenatal clinic (see Figure 1).

### Methods

A qualitative study utilising narrative analysis [27,28] was used. Ten (10) couples aged ≥ 18 years old who underwent couple HIV testing at a public sector antenatal clinic were interviewed. The marital partners were interviewed separately. Maximum-variation sampling [29] was used to select the study participants. This sampling strategy allows for selection of participants with a wide variety of ‘lived’ experiences (and not representative sample) in order to elicit a range of experiences, views and interpretation about a subject matter (in this case couple HIV testing). The women were identified and recruited through an HIV support group based at the antenatal clinic. Since men did not attend HIV support group meetings at the antenatal clinic, initial contact to recruit them was made through their spouses who attended these HIV support group meetings. In addition, five women and two men who had undergone couple HIV testing but were later abandoned by their spouses were also interviewed; their spouses could not be traced. In addition, key informant interviews were held with lay HIV counsellors (n = 5) and antenatal clinic nurses (n = 2) to gain in-depth understanding of the couple HIV testing process and their experiences of providing couple HIV testing.

Face-to-face, open-ended, in-depth interviews were conducted by the lead author. The interviews were conducted between March 2010 and September 2011. Interviews with the counsellors and nurses were conducted in English while interviews with the women and men were conducted in *Nyanja,* the local language widely spoken in the area. The interviews were framed as a narrative production. Research participants were asked to describe their HIV testing experiences as stories, recounting how they came to get tested as couples, what happened inside the antenatal clinic and their ‘lived’ experiences after couple HIV testing. All interviews with clinic staff were conducted at the antenatal clinic while the interviews with couples were conducted outside the clinic setting to ensure that the respondents were not inhibited by the clinic surrounding to share their experiences. All interviews were audio-recorded and usually lasted about 30 minutes. In addition to the interviews, the first author conducted sit-in observations at the antenatal clinic and attended HIV support group meetings based at the antenatal clinic.

All interviews were transcribed verbatim and field observational notes typed. These were imported into, and organised and managed using, Atlas ti version 6. The transcripts and field notes were read several times to develop a deep sense of the data. For interview transcripts, within-case and across-case analysis [30] and ‘paradigmatic analysis’ of narrative [31] were undertaken to inductively generate themes and concepts across the individual narratives [28]. Three reference points were used to identify emergent themes: recurrence, repetition and forcefulness of ideas within the narrative data [32]. For each narrative, we conducted within-case analysis and retrieved and coded each participant’s HIV testing experience. Thereafter, we conducted across-case analysis by comparing and contrasting participants’ experiences. This enabled us to construct storylines across study participants’ testing experiences. By using this analytical strategy, we were able to generate experiences across study participants that were still grounded in individual experiences [30]. Lastly, we selected interview excerpts that best illustrated the storylines.

### Ethical approval

The study was approved by the Ethics Committee of the State of Basel (Ethik-Kommission beider Basel) and the University of Zambia Humanities and Social Sciences Research Ethics Committee within the framework of a bigger research project - ‘Improving equity in access to HIV care and treatment in Zambia.’ Administrative approval was obtained from the Ministry of Health at national and district levels. Written informed consent was obtained from all research participants. To maintain confidentiality, no identifying information is mentioned in the narrative transcripts.

## Results

### Characteristics of study participants

Out of the 10 couples, three (3) were in discordant marital relationships in which the women were HIV-negative (Table 1). In total, ten of the fifteen women and eight of the twelve men were on ART while the rest were not yet clinically eligible for treatment. The respondents were relatively young couples: the majority of the women were in their twenties while the men were in their thirties. The main outcomes of couple HIV testing are summarized in Table 1.

**Table 1:** Characteristics of couples & main outcomes of couple HIV testing

**Age of marital partners (in year)**	**Length of marriage (in years/months)**	**Couple-HIV Status**	**Main outcome of CVCT**
*Couple 1*: Man 32 yrs; woman 28 yrs	4 yrs	Concordant couple	Supportive marriage; protectivesex; woman assertive on condoms. Man provides treatment support.
*Couple 2*: Man 32 yrs; Woman 27 years	4.6 yrs	Concordant couple	CVCT strengthened marital bond; man ‘sticks’ to his wife; alternate use of condoms.
*Couple 3*: Man 36 yrs; Woman 29 years	8 yrs	Concordant couple	Man refuses to use condoms; threatens wife with divorce; wife economically dependent on spouse.
*Couple 4:* Man 26 yrs; Woman 22 years	3.8 yrs	Concordant couple	Man felt “trapped” to test; CVCT empowered wife to form new treatment support social networks.
*Couple 5*: Man 34 yrs; Woman 26 years	2.6 yrs	Concordant couple	Initial cessation of sex after CVCT. Safe sex still a challenge. However, CVCT enabled woman create supportive social support networks.
*Couple 6*: Man 23 yrs; Woman 20 years	2.1 yrs	Concordant couple	Supportive couple; young couple struggling to balance between Protect tion & procreation.
*Couple 7*: Man 34 yrs; Woman 26 years	4.9 yrs	Concordant couple	Man felt “trapped” to test; but encourages wife to attend support group meetings.
*Couple 8*: Man 26 yrs; Woman 22 years	4 yrs	Discordant couple	Supportive couple; reproductive (woman HIV-) aspirations undermine safe sex.
*Couple 9*: Man 28 yrs; Woman 23 years	2.6 yrs	Discordant couple	Strong bond but man’s desire for (woman HIV-) sexual intimacy & child bearing affects safe sex practice.
*Couple 10*: Man 46 yrs; Woman 29 years	5.7 yrs	Discordant couple	Man refuses safe sex; he wants (woman HIV-) male child; HIV-negative woman fears infection & threatens divorce.

### Strategies of promoting couple HIV testing in antenatal clinic

#### Pregnant women directed to bring spouses

Aware of low participation of men and often confronted by fears that some married women faced to test and later disclose their HIV status to their spouses, one strategy, although infrequently and unsuccessfully used by antenatal clinic staff, was to instruct women to bring their spouses for HIV testing. One female lay counsellor explained this process:

“Every day, we test about 30 pregnant women but because men rarely come here, we have adopted a deliberate strategy where half the women are asked to bring their spouses. So for instance, we count 1, 2, 3, 4, 5 up to 15, then the rest, we tell them to go back and come with their spouses.”

Sometimes men were summoned under the pretext of discussing pregnancy and the well-being of the unborn child. Two antenatal clinic staff narrated:

*“There is another option we use. We tell the women that ‘go and tell your husband, there is something that we want to discuss about the baby with him.’ You know when men hear anything to do with the baby, they come….We use that as a chance to counsel them for HIV, test them and tell them their results together.”* (Female nurse, antenatal clinic)

*“He just thinks that may be it has to do with the woman’s pregnancy. When they come here, they are educated about the pregnancy, family planning, STIs, and then they are tested. Normally, when the man is already here, he fails to say I do not want to test.”* (Female lay counsellor, antenatal clinic)

To beguile the men and to motivate the women to bring their spouses for couple HIV testing, women who brought or came with their spouses for HIV testing were given preferential treatment so that the men as breadwinners did not spend a lot of time at the clinic. In the light of congestion at public sector antenatal clinics, women found this strategy of being given preferential treatment appealing and often encouraged their spouses to come for couple HIV testing.

#### Coercing men to test

In some cases, women themselves reportedly used coercive strategies, sometimes threatening not to go for antenatal care and ultimately holding their spouses responsible for any pregnancy-related complications if they refused to accompany them to the antenatal clinic. As one woman explained:

*“I told my husband that if we do not go to the clinic together, I will never go for antenatal care....If anything happens to my pregnancy and my life, my family will hold you responsible. When I said this, he agreed to come with me to the clinic*.” (32-year old woman, living with HIV)

#### Inside the clinic, ‘opt-out’ HIV testing not fully articulated

During group counselling, observations revealed that the opt-out requirement was not explicitly articulated. Antenatal clinic staff often emphasised the bio-medical benefits of testing, including access to treatment and prevention of mother-to-child transmission (PMTCT) of HIV. Sometimes moral obligations were used to encourage uptake of HIV testing. As one lay counsellor said during one group HIV counselling meeting: *“You have to test to protect the child….It is the right of the baby to be born negative, to live a normal and healthy life.”*

By simply evoking maternal/paternal responsibility to encourage uptake of HIV testing, couples were deprived of the right to consent and time and opportunity to reflect on the implications of HIV testing. While women interviewed acknowledged the bio-medical benefits of testing, including for prevention of mother-to-child transmission of HIV, they also expressed concerns about the possible negative impact of testing on their marital relationships. As one woman illustrated:

*“At the clinic, you have little chance of refusing to test. They tell you that you have to do it to protect the child, meanwhile you; you are thinking about what will happen at home. You know a lot of things happen in these marriages.”* (24-year old woman, living with HIV)

Some men reported feeling ‘trapped’ in the antenatal clinic and only acquiesced to test for fear that their partners could be denied antenatal care services. Even though some men reported knowing beforehand that they might end up getting tested, others were oblivious of this possibility. One man elucidated his experience:

*“I really felt trapped when I was told that I needed to test because I did not go to the clinic in order to test. My wife told me that I was wanted so that they can tell us about the pregnancy and how to look after the unborn child.”* (34-year old man, living with HIV)

### Effects of couple HIV testing on marital relationships

#### Couple emotional and social support

For some couples, joint knowledge of their HIV status became a platform to renew their commitment to marriage and family life in the face of HIV, enabling them to be sensitive and responsive to the treatment, emotional and social needs of each other:

*“Me, that is when my marriage became sweet; what can I compare it to? It is as if we have just started our relationship….My husband begun to love me more than before. I don’t know whether he felt guilty, and was a way of compensating for his misdeeds.”* (27-year old woman, living with HIV).

#### Development of supportive social relationships and networks

Outside the household setting, couple HIV testing enabled some women to develop new social network relationships and receive additional social support outside marital relationships. However, this was not found to be the case amongst men as most of them preferred to conceal their HIV status and did not attend HIV support group meetings. One woman narrated her experience:

*“Since both my husband and I tested, I feel free to come for support group meetings, to go to the clinic for my drugs. I now have friends who are also HIV positive. We encourage each other.”* (26 year old woman, living with HIV)

#### Access to and retention in antiretroviral therapy care

Couple HIV testing also helped legitimise access to treatment on account of disclosure of HIV status to marital partner. Couples on HIV treatment gave accounts of how testing as a couple had made it easier for them to access and remain on treatment. For instance, as ‘treatment supporters’, sometimes men collected HIV medication on behalf of their wives or encouraged them not to miss taking their medication. One woman explained:

*“My husband is very supportive. He always reminds me to take my medication. He sets his alarm clock and even when he is not at home, he calls to make sure that I have not forgotten to drink my medicine.”* (28-year-old woman, living with HIV)

Another woman echoed these sentiments:

*“When it is my appointment day at the ART clinic, my husband works up early in the morning, at 04 hours and comes to line up here on my behalf, and I follow later. When it is the time to bring my child for under-five, he even sets the alarm clock to wake me up.”* (26-year old woman, living with HIV)

#### Negotiating safe sex in marital relationships

In the light of HIV-positive test results, negotiating adoption of safe sex was also a recurrent theme among some couples. Our findings revealed that although couples did not always practice safe sex, for some women, being counselled and tested together as a couple had empowered and legitimised their quest to negotiate and demand, or sometimes secretly adopt, safe sexual practices to avoid contracting HIV or re-infecting one another. As one woman poignantly put it:

*“We agreed that we use condoms so that ‘you keep your HIV virus which is used to the medication and I keep my HIV virus which is not yet used to the medication.’”* (27-year old woman, living with HIV)

Our respondents’ narratives further indicate that even for couples that struggled to agree on safe sex, women’s access to female condoms during antenatal care and at support group meetings empowered them to *‘secretly’* protect themselves that “*he [husband] would not even know that I am using the female condom.”* (26-year old woman, living with HIV).

Despite these bio-medical and social benefits, our study also revealed that couple HIV testing had negative effects on marital relationships:

#### Testing space still reproduced entrenched gender inequalities

Although women felt comfortable, even empowered in the public antenatal clinic space, in more intimate spaces, either within counselling sessions or at home, entrenched power relations which sometimes gave rise to strained marital relationships arose. Men were reportedly more assertive during counselling sessions than women, and at home, asserted their authority over women on sexual and reproductive health decisions. One counsellor illuminated her experience:

“Although during counselling session you are not supposed to concentrate on one person, the problem is that men tend to dominate discussions; women feel intimidated…. Most women say ‘I agree with whatever my husband has said.’ It has to do with our culture.”

#### Safe sex or safe marriage?

For both concordant and discordant couples, couple HIV testing did not always lead to practicing safe sex. This was undermined by tension between protection on one hand and the desire for sexual intimacy and procreation on the other hand, sometimes modulated by gendered power relationships. For some couples, having unprotected sex was construed as *sine qua non* for consummating marital relationships while condom use was synonymous with having sex with a non-regular sexual partner - with no emotional intimacy attached. Consequently for some couples, the desire for sexual intimacy, and particularly for women to preserve their marriages, compelled them to engage in unprotected sex, even at the expense of their own health.

Modulated by gendered power relationships in marital relationships as well as economic dependence on their spouses, some women acquiesced to the demands of their spouses in order to preserve their marital relationships. One 29-year old woman in an HIV concordant marriage recounted her experience:

“He told me that if you do not want sex without a condom, then you will go back to your parents and I will find another woman to marry. So since I am scared of losing my marriage, I just give him ‘live’ sex. What can I do because when I tell him about using condoms, he refuses, so what can I do, apart from re-infecting him? Me, I want to help him, and also help myself from getting re-infected, but he threatens to divorce me. How am I going to look after myself and my children?”

Failure to practice safe sex sometimes led to previously HIV-negative partners particularly women getting infected. An ART nurse explained:

“You know when a couple comes, they are discordant, you find that the man is positive and the woman is negative, the woman would support the husband, she will always be with him, and come to the clinic together and what do you discover further, 3–4 months? The wife is also positive. But if a man is negative and the woman is positive, you would find the woman losing weight, the woman coming alone, and the man would never get the virus.”

For discordant couples, negotiating this intricate balance between HIV prevention and preserving one’s marriage was acutely complex and sensitive. A case study illuminates this dilemma: A 46-year old man and his 29-year old wife had been married for almost 6 years. The man had HIV while the woman was not infected. The man insisted on unprotected sex because he wanted another child, a son, while the woman wanted the couple to practice protective sex to avoid getting infected. While she valued her marriage, she threatened to break up with the husband to preserve her HIV-negative status unless the spouse acquiesced to the use of condoms.

#### Strained marital relationships: cessation of sex, mental abuse and abandonment

Another downside of couple HIV testing was the reported cessation of sexual relationships (sometimes intermittent, in some cases permanent), and experiences of mental abuse and abandonment. This was not limited to discordant relationships. One woman whose husband was also HIV-positive explained:

*“What happened was that after testing, my husband shifted from the bedroom and started sleeping in the sitting room. From January - June, my husband slept in the sitting room….”* (34 year-old woman, living with HIV)

Cases of mental abuse, although infrequent, were noted. A counsellor recounted:

“Such cases arise from time to time. I had such an experience before where the man was torturing, ill-treating the wife. The man was negative and the wife was positive. Each time the man came from work, he would tell the wife, ‘come out of the bedroom, you are a sick woman. I have come with another woman.’ Then the woman would sleep in the sitting room, and the man would go into the bedroom with another woman. I felt very bad. I tried to talk to the man but he was a drunkard. He was so sarcastic. So I referred him to another counsellor for further counselling. So I do not know what happened from there.”

Our findings show that separation or divorce took place, often triggered by knowledge of HIV status. This was not restricted to discordant couples only. Three women and two men living with HIV also reported being abandoned by their HIV-positive spouses after couple HIV testing. Two respondents narrated their experiences:

*“We were both tested and found HIV positive but to my surprise, my husband decided to desert me. He just left without saying a word. I later just heard that he was living in […….] (another town). He left me when I was 8 months pregnant. He does not even know the child, he has never seen her, and he has never called me.”* (24-year old woman, living with HIV)

*“It (couple testing) affected our relationship. Her friends started pressurising her to leave our marriage. My wife and her friends opened a saloon at local market, and in no time, she stopped coming home. She started living in [……] compound….I looked for my wife and pleaded with her to come back but she refused.”* (46-year old man, living with HIV)

Due to our inability to interview individuals who had abandoned their spouses after testing, we were unable to establish the reasons for their actions. However, two interviews with two men abandoned by their spouses pointed to blaming attitude as one of the reasons for marriage dissolution.

## Discussion

Our study explored how couple HIV testing was undertaken and its impact on marital relationships. Low male partner participation in antenatal HIV counselling has been reported in other studies [22,33,34]. This was also the case in our study. As a result, our findings suggest that antenatal clinic staff used subtle and sometimes coercive strategies to promote couple HIV testing. This deprived couples not only of voluntary informed consent but also the time and opportunity to weigh the implications of HIV testing. Our findings are consistent with previous studies that have expressed concerns about the impact of ‘opt-out’ provider-initiated HIV testing on voluntary informed consent [35-39] because of the inherent skewed power relationships between health staff and service users. As WHO/UNAIDS have cautioned, ‘endorsement of provider-initiated HIV testing and counselling is not an endorsement of coercive or mandatory HIV testing’ [40].

From the way couple HIV testing was being promoted, our findings suggest that the aim of couple HIV testing was primarily to improve maternal and child health outcomes and not to improve the health status of couples. This explains why some men reported feeling *“trapped”* in the antenatal clinic. Larson and colleagues reached similar conclusions [41].

Contrary to our findings where subtle means were used, previous studies have found that a good proportion of men willingly came for couple HIV testing if sent invitations and were fully counselled on the benefits of testing [23-25]. On the balance, what these findings indicate is that appropriate non-coercive strategies can successfully be implemented and need to be adopted, including adoption of community sensitization campaigns about the value of couple HIV testing.

One notable benefit of couple HIV testing was its positive impact on access to treatment, garnering partner support, adoption of risk reduction sexual behaviour in some instances and the ability by women to form new social network relationships. These findings suggest that being diagnosed with HIV and the uncertainty of living with an incurable infection creates marital cohesion and solidarity. As Rolland [42] has pointed out, a diagnosis of a serious condition (in this case HIV) heightens feelings of loss which can prompt couples either to pull apart or to cling together. Clinging together happens in order to ensure “partnership security” and “relational survival” [43] in the face of HIV. Our study revealed that partner cohesion and solidarity were achieved because some couples accepted the diagnosis, avoided apportioning blame and viewed HIV as a conjoint problem. In terms of adoption of safe sexual practices, we noted that women’s exposure to support group meetings enabled them to be more assertive in marital relationships, including the need for adoption of safe sex to avoid (re-)infection. These findings mirror other studies that have reported increased adoption of safe sex among marital partners that have accessed couple HIV testing [11,12,44].

Given that couple HIV testing facilitated disclosure of HIV status to marital partner, this helped legitimise access and adherence to treatment and access to social support. Women reported being reminded, encouraged and supported by their spouses about taking their medication. Outside the household setting, couple HIV testing provided women with opportunity to develop new social network relationships and receive social support beyond household level. Our findings corroborate other studies which have shown that couple HIV testing increased uptake of ART [13,32,44]. These benefits clearly demonstrate the value of its promotion.

As a caveat, couple HIV testing needs to be promoted while being sensitive to individuals’ or couples’ ‘lived’ experiences given the social context and dynamics of marital relationships. This is especially so given the physical, mental and sexual violence that women are subjected to. For instance, the Zambia Demographic and Health Survey 2007 report indicates that almost half (47%) of women aged 15–49 years interviewed had experienced physical violence in their life time and one-third of women had experienced physical violence in the 12 months preceding the survey; one in five women had experienced sexual violence at some point in their lives [15]. Although experiences of physical violence after couple HIV testing were not reported in our study, however, intermittent and permanent cases of strained marital relationships, including abandonment by spouses after undergoing couple HIV testing were reported. Our findings corroborate previous findings [31,44]. Larson and colleagues have reported that men found couple testing unappealing because of the unstable and distrustful nature of their marriages [41]. These findings demonstrate the fragility of marital relationships.

The findings also suggest that the effects of couple HIV testing on marital relationships may be modulated by inequitable, gendered power relationships. For instance in their familial relationships, women are confronted with patriarchal power dynamics and even those that assert their position in public spaces on HIV seem to have little control over their health when dealing with men in their private lives [45]. In our study, men were reported to be generally more assertive during counselling sessions than women, and at home, asserted their authority over women on sexual and reproductive health decisions. Our findings therefore echo previous calls for gender-sensitive HIV control activities [37,46,47] to avoid adverse social effects.

Couple knowledge of HIV status did not always lead to adoption of risk reduction sexual behaviour. This was linked to socially constructed views of sexual intimacy and the difficulties of balancing between HIV prevention and child bearing aspirations. Men often declined the use of condoms and the subordinate position of women compelled them to acquiesce. Previous studies have reported the challenges of reconciling protection on one hand with childbearing aspirations [48-51] and sexual intimacy [52] on the other hand, thus leading to unprotected sexual behaviour.

The study has both strength and limitations. The findings are based on a relatively small, purposively chosen sample, with a relatively young group of couples who accessed couple HIV testing in one public sector clinic. The findings may therefore not reflect what was going on in other clinics or the experiences of couples in different settings. Further research is needed to encapsulate the experiences of diverse couples receiving provider-initiated couple HIV testing in other settings. More so, interviewing men and women who abandoned their spouses after testing would have provided more insight into factors underlying partner abandonment.

Despite these possible limitations, the findings could still be generalisable to similar settings and clinics that have adopted similar provider-initiated couple HIV testing strategies. What we have attempted to do is to draw on the deep, rich narratives of a small set of couples in order to elicit rich lived experiences of undergoing provider-initiated couple HIV testing to provide insights which can help policies and practices aimed at improving couple HIV testing.

### Lessons for policy and practice

Our findings have implications for better delivery of couple HIV testing services. At policy level, couple HIV testing in antenatal clinics as part of PMTCT needs to be clearly and explicitly articulated within PMTCT protocol, and operational guidelines on how to implement it developed. This will ensure that at service delivery level, couple HIV testing is not conducted simply to meet PMTCT targets but to respond to the health needs of both men and women.

At service delivery level, while efforts to encourage couple HIV testing should be promoted, the ‘opt-out’ requirement needs to be well articulated to ensure that individuals’ rights are respected and couples are given time and opportunity to reflect on the implications of HIV testing. Similarly, non-coercive strategies need to be adopted. Previous couple HIV testing programmes have shown than non-coercive strategies can successfully be implemented [23-25]. Second, even when couple HIV testing is promoted, during counselling sessions, service providers need to be sensitive to the experiences and fragility of marital relationships, including paying close attention to and addressing gendered power relationships. This is because couple HIV testing may be achieved at the expense of harmony in and stability of marital relationships. Third, since one of the primary aims of promoting couple HIV testing in antenatal clinics is to facilitate adoption of protective sexual behaviour [18], counsellors should focus on improving the image of condoms within marriages during counselling sessions to facilitate their use. Fourth, couple participation in support groups should be encouraged and sustained. Efforts should also be made to encourage men to attend support group meetings including scheduling these meetings during weekends when men have time-off from their respective livelihood activities. Fifth, there is a need to make antenatal clinics more male-friendly, including recruiting and using male lay counsellors and nurses and possibly integrating some male health care services in antenatal clinics. This could make men feel comfortable and part of maternal and child health care services. Lastly, given the availability of antiretroviral therapy that significantly reduces the viral load and subsequent transmission of HIV [53,54], closer professional support should be provided to concordant and discordant couples with childbearing ambitions.

## Conclusions

While couple HIV testing is an important HIV prevention strategy, the way it is undertaken needs to be needs-based and beneficiary-responsive. Our study indicates that in its current form, couple HIV testing in antenatal clinic is coercive and subtle, thus undermining informed consent. Couple HIV testing also has negative effects including abandonment, mental abuse and cessation of sexual relationships in some cases. This was despite the enormous bio-medical and social benefits that included access and adherence to treatment and social support, and in some cases adoption of protective sexual behaviour. To build on these benefits, there is need to make couple HIV testing in antenatal settings less coercive, more male-friendly as well as being sensitive and responsive to gendered power relationships and fragility of marital relationships. The tension associated with balancing between HIV prevention and sexual intimacy and procreation amongst concordant and discordant couples needs to be mitigated by improving the image and use of condoms in marital relationships and providing continuous close medical support to HIV concordant or discordant couples with reproductive health aspirations.

## Competing interests

The authors have no competing interests to declare.

## Authors’ contributions

MM conceptualized the study, did the data collection and analysis and drafted the manuscript. VB and SM contributed towards the conceptualization of the study, the analysis and interpretation of the findings and provided input in the drafting of the manuscript. All authors have given final approval of the version to be published.

## Pre-publication history

The pre-publication history for this paper can be accessed here:

http://www.biomedcentral.com/1472-6963/13/97/prepub
